# Cytochemical analysis of cerebrospinal fluid in tuberculous meningitis versus other etiologies

**DOI:** 10.1371/journal.pone.0318398

**Published:** 2025-03-28

**Authors:** Miguel Hueda-Zavaleta, Juan C. Gómez de la Torre, Claudia Barletta-Carrillo, Gustavo Tapia-Sequeiros, Cinthya Flores, Cristian Piscoche, Cecilia Miranda, Ada Mendoza, Marco Sánchez-Tito, Vicente A. Benites-Zapata

**Affiliations:** 1 Diagnóstico, tratamiento e investigación de enfermedades infecciosas y tropicales, Universidad Privada de Tacna, Tacna, Peru; 2 Hospital III Daniel Alcides Carrión – Essalud, Calana, Tacna, Peru; 3 Sequence Reference Lab, San Isidro, Lima, Peru; 4 Facultad de Ciencias de la Salud, Universidad Privada de Tacna, Tacna, Peru; 5 Unidad de Investigación para la Generación y Síntesis de Evidencias en Salud, Universidad San Ignacio de Loyola, Lima, Peru; Stellenbosch University, SOUTH AFRICA

## Abstract

**Background:**

Meningeal tuberculosis (TBM) is the most severe form of extrapulmonary tuberculosis due to its high mortality and long-term sequelae in survivors.

**Methods:**

A cross-sectional study of diagnostic tests was carried out in a private clinical laboratory in Lima, Peru. All cerebrospinal fluid (CSF) samples from patients with suspected meningitis were analyzed with cytochemical and biochemical studies, as well as smear microscopy, India ink, the FilmArray Meningitis/Encephalitis panel, Xpert® MTB/RIF or Xpert MTB/RIF Ultra, and culture for common bacterias, fungi or mycobacterial.

**Results:**

450 CSF samples were included. The main microorganisms detected were *Mycobacterium tuberculosis* (8.9%), *Cryptococcus neoformans* (6.0%), and *Streptococcus pneumoniae* (2.4%). 97.5% of patients with TBM presented positive Xpert MTB/RIF or Ultra. The median number of red blood cells, leukocytes, and percentage of mononuclear cells, glucose, and proteins in the CSF was 57.5 cells/μl, 91.5 cells/μl, 70%, 22.5 mg/dL and 218.3 mg/dL, respectively. Likewise, patients with TMB had the lowest glucose levels (median: 22.5, IQR: 11 - 35) compared to other etiologies of meningitis. While bacterial meningitis had the highest leukocyte (median: 173 μl; IQR: 17 - 520) and protein levels (median: 289.7; IQR: 92 - 556).

**Conclusion:**

The characteristics of the cytochemical study of CSF can guide the differential diagnosis by identifying general trends of tuberculous meningitis and other meningitis etiologies. However, it remains necessary to establish methods with greater precision to properly define the etiological agent causing meningitis.

## Introduction

Meningeal tuberculosis (TBM) is the most common form of tuberculosis of the central nervous system, it represents 5 to 10% of cases of extrapulmonary tuberculosis and has high morbidity and mortality, mainly when there is a delay in the start of treatment. The mortality of TBM has been estimated to be 42.12% (95% CI: 26.46% to 58.53%) [1], which is usually double that observed in meningitis due to other infectious agents [2]. Additionally, co-infection with HIV increases the TBM fatality rate by 53.39% (95% CI: 40.55-66.24) [3]. On the other hand, the neurological sequelae occur in up to 50% of survivors [4].

Although, some clinical characteristics, such as the duration of illness greater than seven days and the presence of moderate pleocytosis with increased proteins (between 100 and 500 mg/dl) and low glucose (less than 45 mg/dl) in the cerebrospinal fluid (CSF) suggest TBM [5], currently TBM is still a diagnostic challenge. This is due to nonspecific symptoms and the difficulty detecting **Mycobacterium* tuberculosis* in CSF. Although culture is the gold standard for diagnosing TBM, it has moderate sensitivity (between 50 and 60%) and requires a long growth time, which delays the diagnosis [6]. Likewise, molecular tests such as Xpert® MTB/RIF require moderate processing complexity and suboptimal sensitivity (~70% sensitivity) for the diagnosis of TBM [7]. Additionally, their high cost compared to conventional tests like smear microscopy makes implementation difficult in low- and middle-income countries [8].

Specific CSF biomarkers for the early and accurate diagnosis of TBM are essential to avoid delays in treatment and reduce the morbidity and mortality associated with this disease. In this context, there is a need to evaluate the differences in CSF cytochemical characteristics to differentiate meningeal tuberculosis from other etiologic agents of meningitis.

## Materials and methods

### Research setting and design

We carried out a cross-sectional study of diagnostic tests. We adhere to the Standards for Reporting of Diagnostic Accuracy Studies (STARD) guidelines for reporting diagnostic test studies [9]. The study was carried out in a private clinical laboratory located in the city of Lima, Peru. The laboratory has ISO 9001 and NTP ISO 15189 quality certification.

### Population

We included all CSF sample results archived in a database from patients with clinical suspicion of meningitis, which were processed in the private clinical laboratory between January 01, 2011 and December 31, 2022. The samples from patients with suspected meningitis were analyzed with cytochemical and biochemical studies, as well as smear microscopy, India ink, the FilmArray Meningitis/Encephalitis panel, Xpert® MTB/RIF or Xpert MTB/RIF Ultra (Cepheid, Sunnyvale, CA, USA), and culture for common bacteria, fungi, and mycobacterial. We accessed the data records on July 5, 2023.

### Sample collection and processing

The collected CSF samples were transported in 3 to 5 sterile cryovials of 2 to 3 ml each, at 2 - 8 ºC, and sent to the laboratory for processing. From these cryovials, the cytological study of CSF was carried out using an automated hematology counter to determine the cell count of red blood cells, leukocytes, and the percentage of mononuclear cells. The biochemical study of CSF for determining glucose and proteins was carried out using the Roche Cobas automated biochemistry equipment. The Gram, India ink, and Ziehl-Neelsen stains were carried out from a cryovial. The cultures of common bacterias, fungi, and mycobacteria were carried out in the culture media MacConkey agar, sheep blood agar, Sabouraud agar, and Lowenstein-Jensen agar medium, as appropriate. In addition, 2 ml of CSF was inoculated into a BACTECTM Plus Aerobic/F blood culture bottle (BioMériux, Durham, NC, USA) to increase profitability. All CSF samples were processed to detect *M. tuberculosis* DNA using the GenXpert system with the Xpert® MTB/RIF cartridge, requiring 0.5 ml of CSF in 1.5 ml of diluent. The sample was considered positive when rpoB gene detection was reported within the valid delta Ct interval. Likewise, since 2018, the multi-pathogen molecular detection panel BioFire® FilmArray® Meningitis/Encephalitis (ME) Panel (BioFire Diagnostics, Salt Lake City, UT, USA), and Xpert® MTB/RIF Ultra (Cepheid Sunnyvale, CA, USA), were added, and which were processed according to the manufacturer’s instructions.

### Statistical analysis

The database of the private clinical laboratory was used as a source document [S1 Dataset]. Statistical analysis was performed with Stata v17.0 software (StataCorp., College Station, TX, USA). The qualitative variables were presented as absolute and relative frequencies and were compared using the Chi2 or Fisher’s exact test according to their expected values. Due to their non-normal distribution, quantitative variables, such as median and interquartile range, were compared using the Mann-Whitney U test or Kruskal Wallis test, as appropriate.

Additionally, we conducted a diagnostic performance analysis to detect TBM. The index test resulted from the CSF cytochemical study (cellularity of leukocytes, red blood cells, percentage of mononuclear cells, glucose, and proteins). We compared it with a reference method composed of the Xpert MTB/RIF or Ultra or culture of *M. tuberculosis* (If either of the two was positive, it was considered positive; if both tests were negative, it was considered a negative test). We investigated the diagnostic capacity of the CSF cytochemical study for TBM using ROC (Receptor Operating Characteristics) curves, the area under the ROC curve (AUC), and the cut-off points selected according to the highest Youden index. In addition, we also determined its sensitivity, specificity, and positive and negative likelihood ratio (LHR).

### Ethics approval and consent to participate

This research follows the Helsinki standards for research on human subjects. The protocol was approved by the Institutional Research Ethics Committee (CIE) of the Private University of Tacna (FACSA-CEI/008-04-2023). Waiver of informed consent was requested due to the retrospective and observational nature of the study. Likewise, the anonymity of each individual was maintained in the collection and cleaning of the database.

## Results

In the present study, we compared CSF characteristics in TBM patients with other meningitis types, including bacterial, viral, and cryptococcal. 450 CSF samples from patients with suspected meningitis were included. The median age was 49 years (IQR: 31 - 69). Pathogenic microorganisms were identified in 22.7% (n = 102) of CSF samples. The main microorganisms detected were *M. tuberculosis* in 40 CSF samples (8.9%), *Cryptococcus neoformans* in 27 CSF samples (6.0%), *Streptococcus pneumoniae* in 11 CSF samples (2.4%), and *Listeria monocytogenes* in 7 CSF samples (1.5%). Only 1.8% of meningitis cases were viral (n = 8), with the Herpes simplex virus (n = 4) and Enterovirus (n = 2) being the main viral microorganisms identified (Table 1).

**Table 1:** Frequency and percentage of main microorganisms detected in cerebrospinal fluid.

Variable	Total (n = 450)
Microorganism not detected Microorganism detected (%)	348 (77.33) 102 (22.67)
- Mycobacterium Tuberculosis	40 (8.89)
- Cryptococcus neoformans	27 (6.00)
- Bacterial - Streptococcus pneumoniae - Listeria monocytogenes - Streptococcus viridans - Enterobacteria - Syphilis - Neisseria meningitidis	27 (6.00) 11 (2.44) 7 (1.55) 3 (0.67) 3 (0.67) 2 (0.45) 1 (0.22)
- Viral - Herpes simplex virus 1/ 2 - Enterovirus - Cytomegalovirus - Human herpesvirus 6	8 (1.78) 4 (0.89) 2 (0.45) 1 (0.22) 1 (0.22)

### Characteristics of CSF in TBM.

Patients with TBM (n = 40) had a median age of 47.5 years (IQR: 34 - 54). 97.5% (n = 39) of them had positive Xpert positive [Xpert MTB/RIF (n = 17) and Xpert MTB/RIF ultra (n = 22)], 27.5% (n = 11) had positive mycobacterial culture, and only 10% (n = 4) had positive Ziehl-Neelsen staining in CSF. Likewise, it was observed that patients with TBM presented a median number of red blood cells, leukocytes, and percentage of mononuclear cells in the CSF of 57.5 cells/μl, 91.5 cells/μl, and 70%, respectively. Regarding the CSF biochemical study, the median glucose and protein were 22.5mg/dL and 218.3 mg/dL, respectively (Table 2). No relevant clinical information was available such as the HIV infection status, duration of illness, clinical presentation, brain imaging, the presence of TB in locations outside the central nervous system.

**Table 2:** Characteristics of the study population and cerebrospinal fluid in patients with meningitis according to etiology.

Variable	Total (n = 450)	Tuberculous meningitis (n = 40)	Cryptococcal meningitis (n = 27)	Bacterial meningitis (n = 27)	Viral meningitis (n = 8)	Microorganism not detected (n = 347)	Valor p
Age (years)^ * ^	49 (31–69)	47.5 (34–54)	50.5 (30.5–50.5)	28.5 (0–40)	19 (18–56)	50 (31–70)	0.137^a^
*Characteristics of CSF*							
Cellularity (cell/μl) ^* ^							
- Red Blood cells	18 (2–198)	57.5 (33.5–134.5)	120 (5–2800)	200 (3–450)	13.5 (1.5–50)	5 (1–120)	<0.001^a^
- Leukocytes	4 (1–70)	91.5 (33–280.5)	10 (2–43)	173 (17–520)	7 (1–132.5)	2 (1–25)	<0.001^a^
- Percentage of mononuclear	80 (40–90)	70 (40–82.5)	80 (45–90)	40 (20–80)	75 (32.5–91)	80 (65–90)	0.019^a^
Biochemical (mg/dl) ^* ^							
- Glucose	56 (41–68)	22.5 (11–35)	35 (14–50)	36.5 (5–57)	63.5 (53–66)	59 (49–71)	<0.001^a^
- Proteins	58.9 (34–140)	218.3 (132.2–276.5)	82 (43.5–126)	289.7 (92–556)	128.2 (39.5–153.7)	47 (30.2–90.9)	<0.001^a^
Positive India ink (%)	26 (5.8)	–	26 (100.0)	–	–	–	–
Positive Ziehl-Neelsen staining (%)	4 (0.9)	4 (10.0)	–	–	–	–	–
Positive gram stain (%)	25 (3.9)	–	–	25 (92.5)	–	–	–
Positive culture - Common bacterias - Cryptococcus	40 (8.9) 18 (4.0) 22 (4.9)	1 (2.50) 1 (2.50) 0 (0.00)	– 22 (81.4)	18 (66.6) –	– –	– –	– –
Filmarray® Meningitis panel positive (n = 249)	45 (10.00)	–					
- Bacteria	18 (4.00)	–	–	18 (66.6)	–	–	–
- Virus	8 (1.8)	–	–	–	–	–	–
- Cryptococcus	19 (4.2)	–	19 (73.3)	–	–	–	–
Positive Mycobacteria culture (%) (n = 211)	11 (2.4)	11 (27.5)	–	–	–	–	–
Xpert® MTB/RIF or Ultra positive (%) - Xpert MTB/RIF - Xpert MTB/RIF Ultra	39 (8.6) 17 (3.8) 22 (4.8)	39 (97.5) 17 (42.5) 22 (55.0)	–	–	–	–	–

* Median and interquartile range; ^a^ Kruskal Wallis test; ^b^ Chi2; CSF: cerebrospinal fluid.

### Differences in CSF characteristics according to etiology

Patients with bacterial meningitis had a higher leukocyte count (median: 173 cells/μl; IQR: 17 - 520) compared to other meningitis etiologies, which was statistically significant. The percentage of mononuclear cells was higher in patients with cryptococcal meningitis (median: 80%; IQR: 45 - 90) and viral meningitis (median: 75%; IQR: 32.5 - 91). Regarding the biochemical study, TBM presented the lowest CSF glucose level (median: 22.5 mg/dl, IQR: 11 - 35), while protein level were highest in bacterial meningitis (median: 289.7 mg/dl; IQR: 92 - 556), followed by TBM (median: 218.3 mg/dl; IQR: 132.2 – 276.5) and viral meningitis (median: 128.2 mg/dl; IQR: 39.5 – 153.7) (Table 2).

### Accuracy of CSF cytochemistry for the diagnosis of TBM

**Fig 1:**
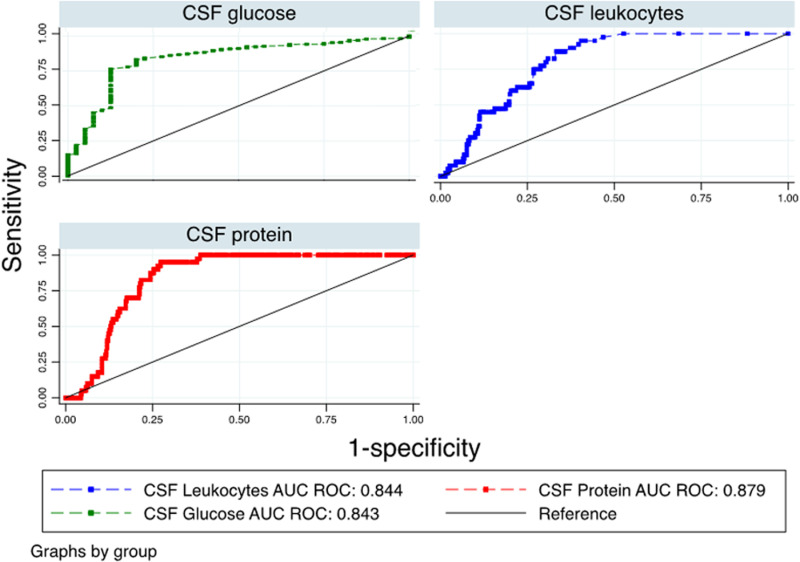
ROC curve of the characteristics of cytochemical study of cerebrospinal fluid to predict tuberculous meningitis.

**Fig 2:**
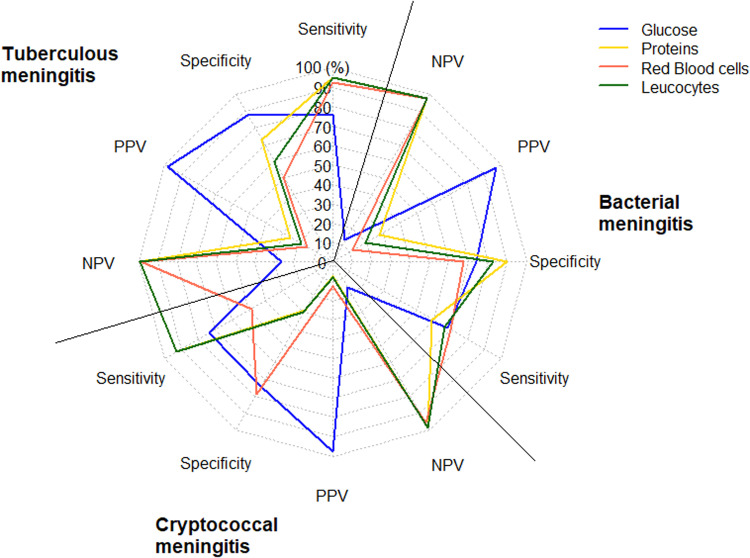
Radar graph summarizing the diagnostic performance of cerebrospinal fluid cytochemical study characteristics.

The cytochemical characteristics of the CSF presented a discriminatory capacity to differentiate TBM from other meningitis. CSF glucose presented an AUC/ROC of 0.84 (95% CI: 0.77-0.90), with a sensitivity of 87.5% and specificity of 76.3% for diagnosing TBM when the glucose level was less than 44.5 mg/dl. The protein level presented an AUC/ROC of 0.84 (95% CI: 0.80 – 0.88), with a sensitivity of 95% and specificity of 72.7% for diagnosing TBM when the protein level was greater than 98.85 mg/dl. Regarding cellularity, pleocytosis greater than 6.5 cells/μl presented a sensitivity of 95%, specificity of 59.5%, and an AUC/ROC of 0.81 (95% CI: 0.76 – 0.85), while the red blood cell counts greater than 6.5cell/μl, presented a sensitivity of 92.5%, specificity of 50.5% and an AUC/ROC of 0.62 (95% CI: 0.56 – 0.69) (Table 3) (Fig 1 and Fig 2)

**Table 3:** Area under the ROC curve and cut-off points with the highest ROC-AUC of cerebrospinal fluid characteristics to predict tuberculous meningitis.

Variable	AUC/ROC	Sensitivity	Specificity	PPV	NPV	LHR ^+ ^	LHR -	Cut-off
Glucose	0.843 (0.769–0.904)	87.50	76.34	26.50	98.40	3.700	0.160	44.50
Proteins	0.879 (0.804–0.880)	95.00	72.68	25.30	99.30	3.478	0.069	98.85
Red Blood cells	0.624 (0.559–0.690)	92.50	50.49	15.40	98.60	1.868	0.148	6.50
Leucocytes	0.844 (0.757–0.854)	95.00	59.51	18.60	99.20	2.346	0.084	6.50
Mononuclears	0.433 (0.337–0.528)	100.00	8.60	19.50	100.00	1.090	0	8.00

AUC: area under the curve; ROC: receiver operating characteristic; PPV: Positive predictive value; NPV: Negative predictive value; LHR: Likelihood ratio; IC 95%: 95% confidence interval.

## Discussion

In the present study, while CSF cytochemical characteristics demonstrated some discriminatory capacity between TBM and other types of meningitis, they were not sufficient to differentiate TBM with high specificity. CSF glucose and protein levels showed moderate accuracy and high sensitivity. Although these findings are promising, they do not surpass the performance of the composite reference method (Xpert MTB/RIF or Ultra with mycobacterial culture). The WHO recommends using Xpert MTB/RIF or Xpert MTB/RIF Ultra as the initial diagnostic test in patients with suspected TBM over smear or culture [10,11]. This is due to the low sensitivity of smear microscopy and mycobacterial culture (between 20 to 40% and 50 to 60%, respectively). Also, the culture requires a prolonged growth time, which could delay diagnosis [6], and lead to worse outcomes due to delayed treatment initiation.

The sensitivity of diagnostic tests may change depending on the reference standard used [12]. A study that evaluated the diagnostic performance of Xpert MTB/RIF and Xpert Ultra found that when the reference standard was only mycobacterial culture, the sensitivity of Xpert MTB/RIF ultra and Xpert MTB/RIF was 90.9% and 81.8%, respectively. On the other hand, when the reference standard was definite, probable, and possible tuberculous meningitis (Score by Marais et al.), the reported sensitivity of the Xpert MTB/RIF ultra was 47.2% and 39.6% for the Xpert MTB/RIF (p = 0.56), which is relatively low to use the Xpert as a TBM rule-out test [13].

Given the limitations of available diagnostic techniques. A group of experts established a consensus on the definition of TBM for its uniform use in research, categorizing the detection of *M. tuberculosis* by baciloscopy, culture, or molecular biology as a definitive diagnosis. The Marais criteria should be used as the reference standard for TBM diagnosis, as is commonly expected in TBM diagnostic studies. The Marais scoring system classifies TBM as either definite or probable TBM, based on clinical criteria, CSF findings, and, optionally, brain imaging [14]. The system proposed by Marais ≥ 12 had an acceptable diagnostic accuracy with an AUC ROC of 0.74, a sensitivity of 50%, and a specificity of 89.3%, using a positive result in smear microscopy, culture, or PCR as the reference. Despite this, it was not useful in distinguishing TBM from other non-TBM etiologies such as fungi, viruses, bacteria, and unknown agents [15]. In our study, we compared diagnostic performance against definite TBM, but it is also important to consider probable TBM to fully evaluate diagnostic accuracy.

Usually, the CSF of patients with TBM has a clear appearance (80 - 90%), hypoglycorrhachia (glucose in CSF < 40mg/dL), proteinorrachia between 50 and 300mg/dL, and pleocytosis between 5 to 1000 cells/ul with a predominance of mononuclear cells ( > 50%). In patients with HIV, the same CSF characteristics are usually observed, although lower CSF leukocyte counts may be observed. These alterations in the CSF are generally present up to 10 to 14 days after starting treatment [16].

One of the parameters with the best diagnostic performance for TBM was CSF glucose, with a sensitivity higher than that reported in other studies, both in patients with or without HIV infection (sensitivity of 68% and 69, respectively). However, our study did not include specific information on HIV status, which may have limited our ability to assess diagnostic performance in this subpopulation. However, there are case reports of TBM with normal CSF glucose, so its absence should not be exclusive [17,18]. Our study reported that a CSF glucose and protein value < 44.50 mg/dL and > 98.85 mg/dL, respectively, have the best discriminatory capacity to identify cases of TBM. A Peruvian study determined that a cut-off point ≤  50 mg/dl for glucose and > 45 mg/dl for protein in CSF were the best indicators of TBM [19]. However, it should be considered that in this study, only 30% of cases are confirmed bacteriologically as TBM. Likewise, our study reported similar glucose cut-off points to identify TBM and bacteria, coinciding with another study that did not find significant differences in CSF glucose for both etiologies, so it should not be used to differentiate them [20]. The increase in CSF proteins was predominant in bacterial meningitis (289.7 mg/dl) and TBM (218.3 mg/dl). According to the literature, there is a marked increase in proteins in both etiologies. Therefore, it is not a parameter to consider to differentiate them [21].

According to PAHO parameters, leukocyte values must exceed ten cells/μl with a predominance of polymorphonuclear cells for suspected bacterial meningitis [22]. Our findings found that a pleocytosis level greater than 6.5 cells/μl had a good discriminatory capacity to identify TBM cases. In comparison, the cut-off point for bacterial meningitis should have exceeded 107.5 cells/μl. This difference may be due to the different criteria defining a positive TBM case (clinical criterion vs. microbiological confirmation).

This study has certain limitations. The main limitation of the study was its retrospective nature from a database, which prevented the assessment of other relevant variables, such as the duration of illness, clinical presentation, brain imaging, or the presence of TB in locations outside the central nervous system. Therefore, in those samples without a definitive diagnosis, it was not possible to determine probable TBM using methods like the Marais et al. score. Another limitation of this study is that the HIV infection status or CD4 counts was unknown, which could have different CSF characteristics and lower sensitivity of the Xpert MTB/RIF. Another limitation is the use of FilmArray as the only test for viral pathogens, which has lower sensitivity for diagnosing HSV-1 compared to conventional PCR (84% vs 99%). Similarly, we did not use cryptococcal latex antigen testing, though we did use a combination of culture, India ink, and FilmArray for cryptococcal detection in the diagnosis of cerebral cryptococcosis. Finally, 100% of the CSF samples were cultured using Lowenstein-Jensen instead of the MGIT system, which has been shown to have greater sensitivity for the detection of M. tuberculosis.

Future studies should focus on populations without mycobacterial confirmation to assess the true value of these biomarkers in broader clinical settings, as mentioned in our limitations.

## Supporting information

S1 Dataset Clinical laboratory database. (XLSX)
